# FACILITATORS AND BARRIERS TO IMPLEMENTING A VIRTUAL REALITY PROGRAM IN LONG-TERM CARE

**DOI:** 10.1093/geroni/igad104.1054

**Published:** 2023-12-21

**Authors:** Lillian Hung, Joey Wong, Mona Upreti, Winnie Kan, Alisha Tumar, Sonia Hardern, Jim Mann, Christine Wallsworth

**Affiliations:** Vancouver General Hospital, Vancouver, British Columbia, Canada; University of British Columbia, Vancouver, British Columbia, Canada; UBC IDEA lab, Vancouver, British Columbia, Canada; University of British Columbia, Vancouver, British Columbia, Canada; UBC IDEA lab, Vancouver, British Columbia, Canada; Vancouver Coastal Health, Vancouver, British Columbia, Canada; Community Engagement Advisory Network, Vancouver, British Columbia, Canada; Vancouver, British Columbia, Canada

## Abstract

To successfully implement virtual reality (VR) programs for long-term care (LTC) residents, it is essential to consider contextual factors. However, current research does not explore the LTC staff’s perspectives on implementing VR in their workplaces. This qualitative study aimed to fill this gap by exploring the facilitators and barriers to adopting VR in LTC, guided by the Consolidated Framework for Implementation Research (CFIR). We applied a Collaborative Action Research (CAR) approach, which involved three phases: (1) Reflect and Plan, (2) Act and Adapt, and (3) Evaluate. Ten focus groups were conducted with 20 staff in two Canadian long-term care homes. Thematic analysis was performed collectively with the team, including researchers, trainees, and patient and family partners. Our findings suggest that implementing a VR program in LTC requires readiness and capacity for implementation within the care home. Key factors that enabled implementation were staff champions, perceived benefits, and ease of use of the equipment. However, there were also barriers, such as limited resources, including Internet infrastructure, limited adaptability to meet local needs, and relative priority and staff workload. To overcome these barriers, our results indicate a need for organizational support for infrastructure and human resources. In addition, future research can evaluate the potential impact of facilitating residents’ VR sessions on staff’s job satisfaction and the involvement of residents’ families/caregivers as well as volunteers during the sessions to reduce staff hesitancy and workload.

